# Understanding the prevalence of mental imagery, music, and their combined use among athletes and coaches

**DOI:** 10.3389/fspor.2025.1683432

**Published:** 2025-10-13

**Authors:** Fernando Castellar, Rocco Cavaleri, Steffen A. Herff

**Affiliations:** ^1^ The University of Sydney, Sydney Conservatorium of Music, Sydney, NSW, Australia; ^2^ Western Sydney University, The MARCS Institute for Brain Behaviour and Development, Penrith, NSW, Australia; ^3^ Brain Stimulation and Rehabilitation lab, School of Health Sciences, Western Sydney University, Sydney, NSW, Australia

**Keywords:** mental imagery, music interventions, combined strategies, mental skills training, cognitive strategies in sport

## Abstract

**Introduction:**

Both mental imagery and music have significant impacts on motor skill acquisition and improvement, as well as on the regulation of psychological factors crucial for athletic success. However, research on the prevalence of these mental practice strategies across sporting domains, as well as their combined application (hereafter reported as MIMUS), is limited. It therefore remains unclear which strategies athletes and coaches adopt in an athlete's mental preparation routine. Accordingly, the present study investigated the prevalence of music, mental imagery, and their combined use among coaches and athletes across different performance levels and sports settings. The content and motivations underlying the use of these strategies were also explored.

**Design and methods:**

One hundred and thirty-six individuals (89 men, 42 women, 5 non-binary, *M_age_* = 35.22, *SD_age_* = 15.82, *Range* = 18–83 years) completed a 23-item online questionnaire regarding athletes' mental preparation regimes in sports. After providing demographic information, participants responded to closed-ended and multiple-choice questions concerning their use of mental imagery, music, and MIMUS, as well as the reasons, contexts, and circumstances (why, how, where, when) in which these strategies were employed.

**Results:**

Bayesian Mixed Effects models showed that mental imagery (MI, 62%), music (MUS, 66%), and their combination (MIMUS, 27%) were all commonly used by the respondents, though athletes and coaches employed them with distinct motivations. While athletes favoured the use of music, coaches more commonly recommended the use of mental imagery to their athletes. At the international level, the use of all strategies increases, especially MIMUS.

**Conclusions:**

The findings highlight that music and mental imagery form a core part of the mental training routine in athletes, and that their use is more prevalent at the top end of the expertise spectrum.

## 1 Introduction

Over the last few decades, research has been conducted to understand the mechanisms underlying expertise development in areas such as sports, medicine, physics, and the arts (3, 4). A key area of interest in expertise development is the role of practice, defined as purposeful and highly structured behavioural regimes aimed at improving domain-specific performance (3). The time an individual engages in these regimes often extends over years and tends to be monotonically related to their acquired performance levels. That is, the time spent training to achieve a skill is a key predictor of one's performance level (5). At the highest levels of sport, however, the intense physical demands of standardised training place an upper limit on the benefits of additional physical practice, as overtraining increases the risk of injury (6–8). In elite competition, where marginal gains can determine success, it becomes essential to incorporate non-physical methods into training. Mental imagery offers one such approach—an effective form of deliberate practice shown to enhance motor performance without adding physical load (1, 9).

Mental imagery regulates key psychological and psychophysiological elements in sports, including arousal, valence, flow, and anxiety (10), enhances muscular activity and strength (11), and increases cortical excitability in relevant brain areas for motor outputs (12, 13). The latter shapes the mental representations of the action, which are stored in long-term memory and transferred into changes in overt stages of an action (14). Altogether, adding mental imagery to a training programme promotes better motor skill performance outcomes compared to physical practice alone (1, 14).

Despite its reported effectiveness in sports literature, there is limited evidence on the extent to which mental imagery strategies are used in ecologically valid settings. To our knowledge, no study has systematically explored the prevalence of mental imagery strategies among a sample of athletes and coaches. In this context, references to coaches' use of mental training strategies relate to how coaches encourage their athletes to employ them in mental training routines, rather than for the coaches' own performance. In, possibly, the only study that provides a subtle hint, Mizuguchi et al. (15) stated that 70%–90% of elite athletes use motor imagery to improve their motor skill performance; however, further details are not disclosed (15). The most consistent finding in the imagery usage literature is that higher-level athletes use imagery more frequently than lower-level ones, demonstrating the role of imagery in sports expertise (16–18). Further, imagery use does not seem to differ between gender (16, 19). Beyond these findings, most research has focused on the contextual and functional aspects of imagery, such as its purpose, timing, content, and delivery, rather than on its prevalence (20). Research on coaches' encouragement of imagery use by athletes is especially limited, highlighting the need to examine this group. Investigating the prevalence of imagery use among both athletes and coaches could reveal differences in how these key stakeholders engage with mental imagery as a deliberate practice in sport.

Integrating music into mental imagery has been proposed as a way to enhance its effectiveness and refine its value as a deliberate practice (21, 22), as the use of music alone in sports increases affective valence, oxygen consumption, motor skill performance, perceived effort, and physical activity adherence (2, 23). In addition, music strongly influences the vividness, themes, sentiment, and temporal-spatial properties of goal-directed mental imagery content (24–27).

In terms of prevalence, music is commonly used by athletes for mood regulation [45% of the sample (28)] and for increasing vigour prior to competition [31% of the sample (23)], although the motives of which coaches suggest their athletes to listen to music have never been reported. Overall, it seems that adding music to mental imagery is a promising avenue to produce a robust synergistic effect and enhance motor skill performance even further.

The efficacy of mental practice strategies combining mental imagery and music (henceforth: MIMUS) has been the scope of studies in the sports performance field, including modalities such as basketball (29, 30), dart-throwing (21), and soccer (22). A recent systematic review (31) included these and other studies that measured the efficacy of MIMUS on motor skill performance improvement. In sum, their review reported strong evidence that MIMUS is an effective tool for mental training aimed at motor skill performance enhancement when comparing pre- vs. post training performance, and promising prospects that MIMUS may also be even more effective than imagery alone. However, little research to date has examined whether, or to what extent, MIMUS is used in applied settings such as training or competition. Thus, the current study aims to (1) investigate the prevalence of mental imagery, music, and MIMUS among a sample of athletes and coaches, and explain the variation in prevalence between these groups; and (2) identify the content and contexts in which they are used, including how, when, why, and where the sample usually apply these mental training practices. Based on the literature described so far, the present study has formulated the following hypotheses:

1. International-level athletes will report more frequent use of all mental practice strategies compared to athletes of lower-tier competitive levels, reflecting their established role in expertise development.
2. Athletes will report a higher prevalence of both mental imagery and music use compared to coaches, given that most existing evidence focuses on athletes, and little is known about coach engagement.
3. Given the strong efficacy evidence in experimental studies (21, 22, 29, 30), but little ecological evidence, we hypothesise that MIMUS use will be less prevalent than imagery or music alone, but still present among both athletes and coaches.

Understanding how mental imagery, music, and their combination are integrated into an athlete's training regime can provide insights into the prevalence of these strategies and their potential impact on performance, informing both research and applied sports psychology.

## 2 Methods

### 2.1 Participants and recruitment procedure

The survey was distributed via email to 592 sports clubs, regional and national organizations across a wide range of sporting modalities (full list of sports available in Supplementary Material S1) located in English-speaking countries, including Australia, New Zealand, Canada, the United States of America, the United Kingdom, Ireland, and Malta. In addition, Australian university-based sporting clubs were contacted. A participant information sheet was provided at the beginning of the survey. Participation was voluntary and all participants provided informed consent. All responses were collected through a Qualtrics survey (Qualtrics, Provo, UT).

To enhance response rates, the authors distributed personalised invitations to clubs emphasising the applied relevance of the study, and reassuring participants of anonymity. Three months after the first e-mail was sent out to the club/organization, a follow-up was conducted and the research team issued reminder emails to non-responding organisations. The authors were available for e-mail consultation in case participants had any queries related to the survey. All data collected was stored in the leading author's Qualtrics (Qualtrics, Provo, UT) account. Once data collection was finished, an excel spreadsheet (32) was downloaded and subsequent data analysis was performed in R4.3.1 (33). The study was approved by Western Sydney University human research ethics committee (approval code: H15799) and conducted in accordance with the Declaration of Helsinki.

### 2.2 Instruments

The questionnaire consisted of questions related to demographic information, including age, gender, the individual's role (athlete or coach), the sport in which they compete, and their competitive level (international, national, regional, and recreational [also see (16)]. Following these questions, participants responded to closed-ended questions (yes or no) indicating whether they used strategies such as mental imagery, music, and MIMUS as part of their (or their athletes) mental training routine. Furthermore, the questionnaire used branching logic, whereby participants either answered further questions about their use of the strategy (i.e., why, how, what type, where, and when they used mental imagery if they responded yes to the question “do you use mental imagery as part of your training routine?”). On the other hand, the survey would skip these questions if participants indicated no use of the strategy. Finally, participants responded to multiple-choice questions related to their use of other mental training strategies, including self-talk, relaxation, meditation, action observation, or none of them. Whenever the most applicable option was not listed in the multiple-choice responses, participants were allowed to type their response in a free-text box. The survey was designed by the research team for descriptive purposes aligned with the study's objectives rather than psychometric testing*.* The full questionnaire is available in Supplementary Material S2.

### 2.3 Statistical analysis

Data analyses were carried out with Bayesian Mixed Effects models using the brms package in R (34), whereby usage of a mental training strategy was predicted by the interaction between responses provided (the use or not of mental imagery, music, and MIMUS), role (athlete or coach), and competitive level (international, national, regional, recreational), whilst controlling for random effects of sport modality. All models were provided with a weakly informative prior (a t-distribution with a mean of 0, a standard deviation of 1, and 3 degrees of freedom (35)), and ran with 1,000 warm-ups and 10,000 iterations on four chains. Then, the marginal_effects package (36) was used to draw posterior predictions and assess whether our variables of interest significantly influenced model predictions through hypothesis testing. For each hypothesis, effect coefficients (β) relevant to the specific hypotheses, the estimated error of these coefficients (EEβ), as well as the evidence ratio in favour of a given hypothesis (Oddsβ), and the Posterior Probability of the effect (Post.Prob) were reported. Effects were considered significant under an evidence ratio ≥19 for one-sided hypothesis tests, and >39 for two-sided hypothesis tests (37). The R script for the model is available in the online supplement available at the OSF entry https://osf.io/xmw7e/.

## 3 Results

One hundred and thirty-six athletes and coaches (89 men, 42 women, 5 non-binary, *M_age_* = 35.22, *SD_age_* = 15.82, *Range* = 18–83 years) responded to the survey. Table 1 shows the demographic information of participants and the distribution across gender, years of experience in their sport modality, hours of practice per week, expertise level, and type of sport practiced.

**Table 1:** Demographic details of participating athletes of the survey.

Role	Variable	Count	Mean	%	Range	SD
	Gender and Age					
Athlete	Female	35	31.28	31.25%	18–74	15.8
Athlete	Male	72	33.88	64.29%	18–83	15.06
Athlete	Non-binary	5	34.5	44.60%	19–69	23.4
Athlete	Total	112	33.08	100.00%	18–83	15.5
	Years of Experience					
Athlete	1 year	11		9.82%		
Athlete	2 to 5 years	34		30.36%		
Athlete	5 to 10 years	16		14.29%		
Athlete	More than 10 years	51		45.54%		
Athlete	Total	112		100.00%		
	Hours of practice per week					
Athlete	1 to 5 h	37		33.04%		
Athlete	6 to 10 h	38		33.93%		
Athlete	11 to 15 h	22		19.64%		
Athlete	16 to 20 h	7		6.25%		
Athlete	21 to 30 h	7		6.25%		
Athlete	31 to 40 h	0		0.00%		
Athlete	More than 40 h	1		0.89%		
	Total	112		100.00%		
	Expertise Level					
Athlete	International	38		33.93%		
Athlete	National	20		17.86%		
Athlete	Regional	33		29.46%		
Athlete	Recreational	21		18.75%		
	Total	112		100.00%		
	Type of Sport					
Athlete	Endurance and Athletics (e.g., Rowing, Track and Field, Swimming, Ice-skating)	16		14.29%		
Athlete	Team Ball Sports (Soccer, Handball, Basketball, Volleyball)	61		54.46%		
Athlete	Combat and Martial Arts (Kendo, Taekwondo, Martial Arts)	21		18.75%		
Athlete	Precision Sports (Bowling, Archery, Golf, Darts)	12		10.71%		
Athlete	Aesthetic & Strength-Based (Powerlifting, Bodybuilding, Artistic Gymnastics)	2		1.79%		
Athlete	Total	112		100.00%		
	Gender and Age					
Coach	Female	7	44.57	29.17%	20–66	17.51
Coach	Male	17	45.23	70.83%	25–66	12.32
Coach	Non-binary	0	0	0.00%		
	Total	24	45.04	100.00%	20–66	13.63
	Years of Experience					
Coach	1 year	2		8.33%		
Coach	2 to 5 years	4		16.67%		
Coach	5 to 10 years	7		29.17%		
Coach	More than 10 years	11		45.83%		
	Total	24		100.00%		
	Hours of coaching per week					
Coach	1 to 5 h	8		33.33%		
Coach	6 to 10 h	7		29.17%		
Coach	11 to 15 h	4		16.67%		
Coach	16 to 20 h	3		12.50%		
Coach	21 to 30 h	0		0.00%		
Coach	31 to 40 h	1		4.17%		
Coach	More than 40 h	1		4.17%		
	Total	24		100.00%		
	Expertise Level					
Coach	International	9		37.50%		
Coach	National	3		12.50%		
Coach	Regional	7		29.17%		
Coach	Recreational	5		20.83%		
	Total	24		100.00%		
	Type of Sport					
Coach	Endurance and Athletics (e.g., Rowing, Track and Field, Swimming, Ice-skating)	4		16.67%		
Coach	Team Ball Sports (Soccer, Handball, Basketball, Volleyball)	13		54.17%		
Coach	Combat and Martial Arts (Kendo, Taekwondo, Martial Arts)	5		20.83%		
Coach	Precision Sports (Bowling, Archery, Golf, Darts)	0		0.00%		
Coach	Aesthetic & Strength-Based (Powerlifting, Bodybuilding, Artistic Gymnastics)	2		8.33%		
		24		100.00%		

### 3.1 Overall, mental imagery, music, and MIMUS are commonly used

A Bayesian Mixed Effect model showed that mental imagery (MI, 62%), music (MUS, 66%), and their combination (MIMUS, 27%) are all commonly used by the participants. The model, however, revealed weak evidence that music is used more frequently than mental imagery in general: MUS > MI [*β* = 0.03, *EEβ* = 0.05, *Odds* (*β* > 0) = 1.37, *Post.Prob =* 0.7]. Importantly, given the branching logic of the survey, the question “Do you use mental imagery and music combined as part of your (your athletes') training routine?” was only displayed when both questions related to the use of music and mental imagery were positive. This means that the reported use of MIMUS can only be equal or lower than the use of music and imagery. Nevertheless, out of a total sample of 55 participants who responded yes to using music and mental imagery, 34 also indicated the use of these two mental training strategies combined, representing 62% of that subset sample.

### 3.2 Mental imagery strategies are more employed by coaches, and music is more commonly used by athletes

#### 3.2.1 Athletes

**Figure 1:**
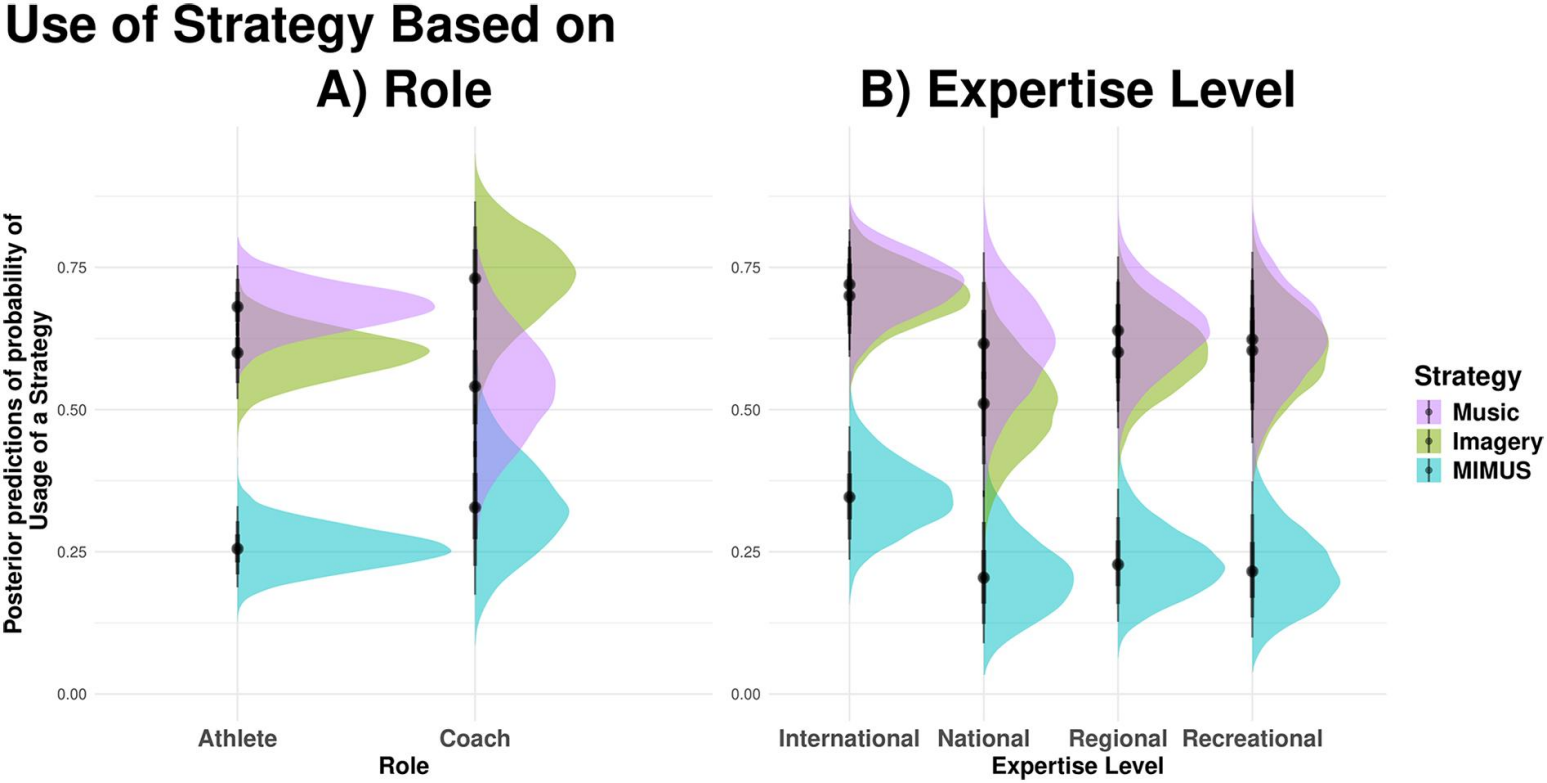
Posterior prediction of probability of usage of a strategy per **(A)** role and **(B)** expertise levels of participants.

The model shows a higher prevalence of music usage than mental imagery and MIMUS by athletes (MI, 60%; MUS, 68%, MIMUS, 26%). The evidence for greater music use compared to MIMUS was strong [*β* = 0.40, *EEβ* = 0.06, *Odds* (*β* > 0) = 9,999*, *Post.Prob =* 1.00], but only moderate when comparing music usage over mental imagery [*β* = 0.40, *EEβ* = 0.06, *Odds* (*β* > 0) = 11.76 *Post.Prob =* 0.92]. Further, there is also strong evidence for a more common use of mental imagery compared to MIMUS by athletes [*β* = 0.35, *EEβ* = 0.05, *Odds* (*β* > 0) = 9,999*, *Post.Prob =* 1.00]. Figure 1A shows the posterior predictions of the prevalence of each strategy across athletes and coaches.

#### 3.2.2 Coaches

Coaches more often encourage their athletes to incorporate mental imagery into their training regimes compared to music (MI, 71%; MUS, 45%, MIMUS, 28%). The model shows strong evidence for a higher prevalence of mental imagery compared to music [*β* = 0.26, *EEβ* = 0.10, *Odds* (*β* > 0) = 147.15*, *Post.Prob =* 0.99] as well as MIMUS [*β* = 0.43, *EEβ* = 0.10, *Odds* (*β* > 0) = 9,999*, *Post.Prob =* 1.00] in the coach cohort. Additionally, there is moderate evidence that coaches recommend music more frequently than MIMUS [*β* = 0.17, *EEβ* = 0.11, *Odds* (*β* > 0) = 17.87, *Post.Prob =* 0.95].

### 3.3 All strategies show greater use at the top end of the expertise spectrum

The comparison of mental practice strategy uses across expertise level of participants (International, National, Regional, and Recreational) yielded similar patterns of response, with greater use of all strategies at the international level. In most pairwise comparisons, the model shows moderate to high evidence of higher prevalence of the use of each strategy by participants at the International level (MI, 70%; MUS, 71%; MIMUS, 35%) compared to National (MI, 48%, Odds = 241.81*; MUS, 59%, Odds = 7.84; MIMUS, 17%, Odds = 38.41*), Regional (MI, 61%; Odds = 8.94; MIMUS, 22%, Odds = 17. 52), and Recreational (MI, 61%, Odds = 7.73; MIMUS, 20%, Odds = 22.74*). However, usage of the three strategies was comparable across the three less competitive levels: recreational, regional, and national (all Odds <10, details on all comparisons can be found in the online supplement OSF). Figure 1B depicts the usage of the mental training strategies based on expertise levels.

### 3.4 Strategy usage is comparable between men and women

**Figure 2:**
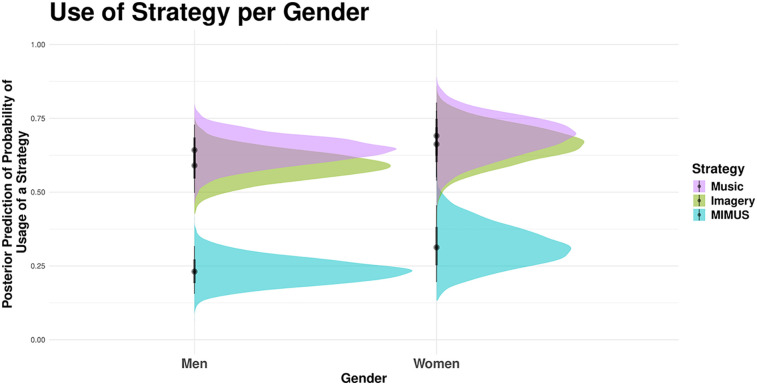
Posterior prediction of probability of usage of a strategy per reported gender.

Another Bayesian Mixed Effect model was used to predict the usage of a mental practice strategy by the interaction between strategy (the use or not of mental imagery, music, and MIMUS) and self-reported gender (women = 42, men = 89, non-binary = 5), whilst controlling for random effects of sport modality. Note, due to the small sample of non-binary respondents, their results could not be reliably statistically interpreted, and the group was not included in the model. However, the data is still available in the online supplement. The model showed only moderate evidence that women use mental imagery [*β* = 0.10, *EEβ* = 0.07, *Odds* (*β* > 0) = 13.55, *Post.Prob =* 0.93] and MIMUS [*β* = 0.11, *EEβ* = 0.07, *Odds* (*β* > 0) = 16.17, *Post.Prob =* 0.94] more commonly than men. There was weak evidence that women used music more often than men (All Odds Ratios < 10). Figure 2 depicts the usage of the mental training strategies based on self-reported gender.

### 3.5 Descriptives of timepoints, motives, and content of mental imagery, music, and MIMUS use

In addition to the prevalence of the use of the strategies, the survey also asked questions related to the contexts (timepoints, motives, and contents) of the use of mental imagery, music, and MIMUS. Below, we report descriptive data regarding these measures. We did not perform statistical analyses on these outcomes as they were considered exploratory in nature.

#### 3.5.1 Timepoints

**Figure 3:**
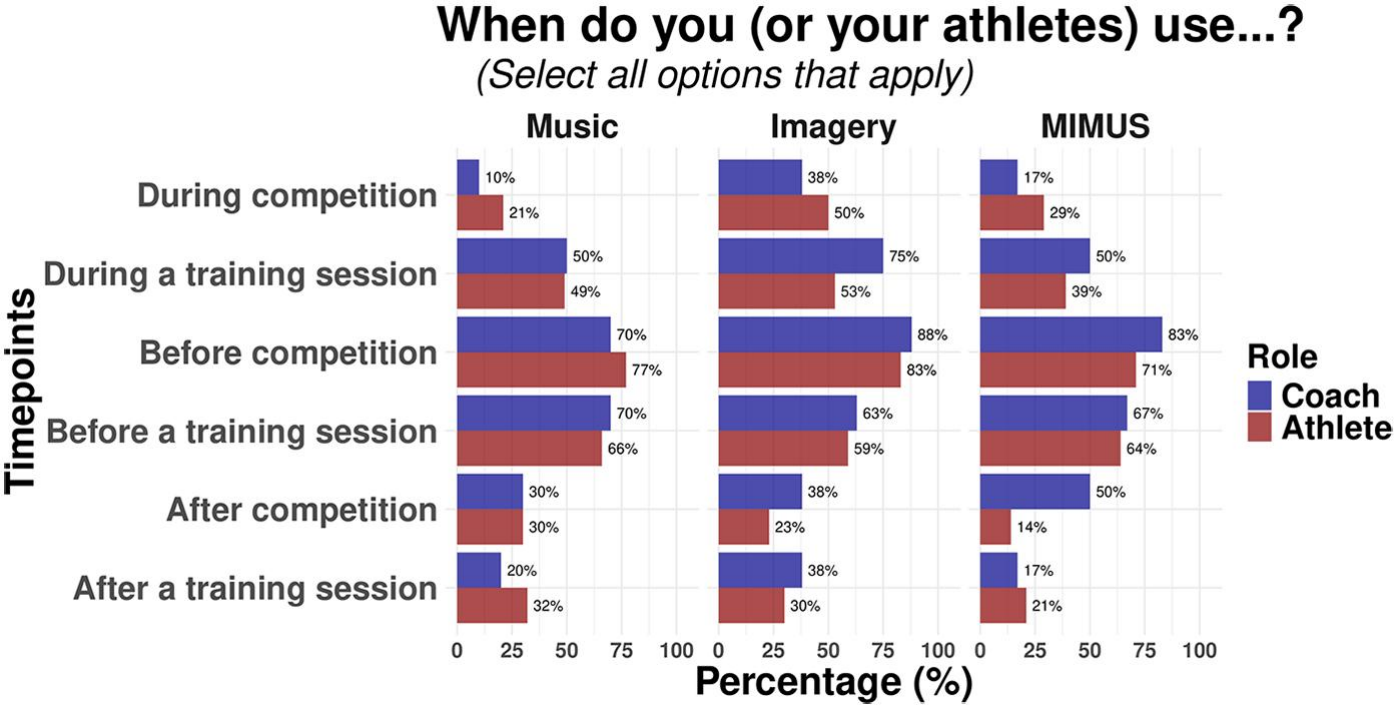
Timepoints when coaches and athletes use music, mental imagery, and MIMUS.

In terms of timepoints, athletes and coaches reported a high frequent use of any strategy before competition (Athletes: MI, 83%; MUS, 77%; MIMUS, 71%; Coaches: MI, 88%; MUS, 70%; MIMUS, 88%) and before a training session (Athletes: MI, 59%; MUS, 66%; MIMUS, 64%; Coaches: MI, 63%; MUS, 70%; MIMUS, 63%). In addition, the use of mental imagery and MIMUS are also highly recommended by coaches during their athletes' training session (MI, 75%; MIMUS, 75%). Figure 3 shows the percentage of participants using each strategy during different timepoints across participants.

#### 3.5.2 Motives

**Figure 4:**
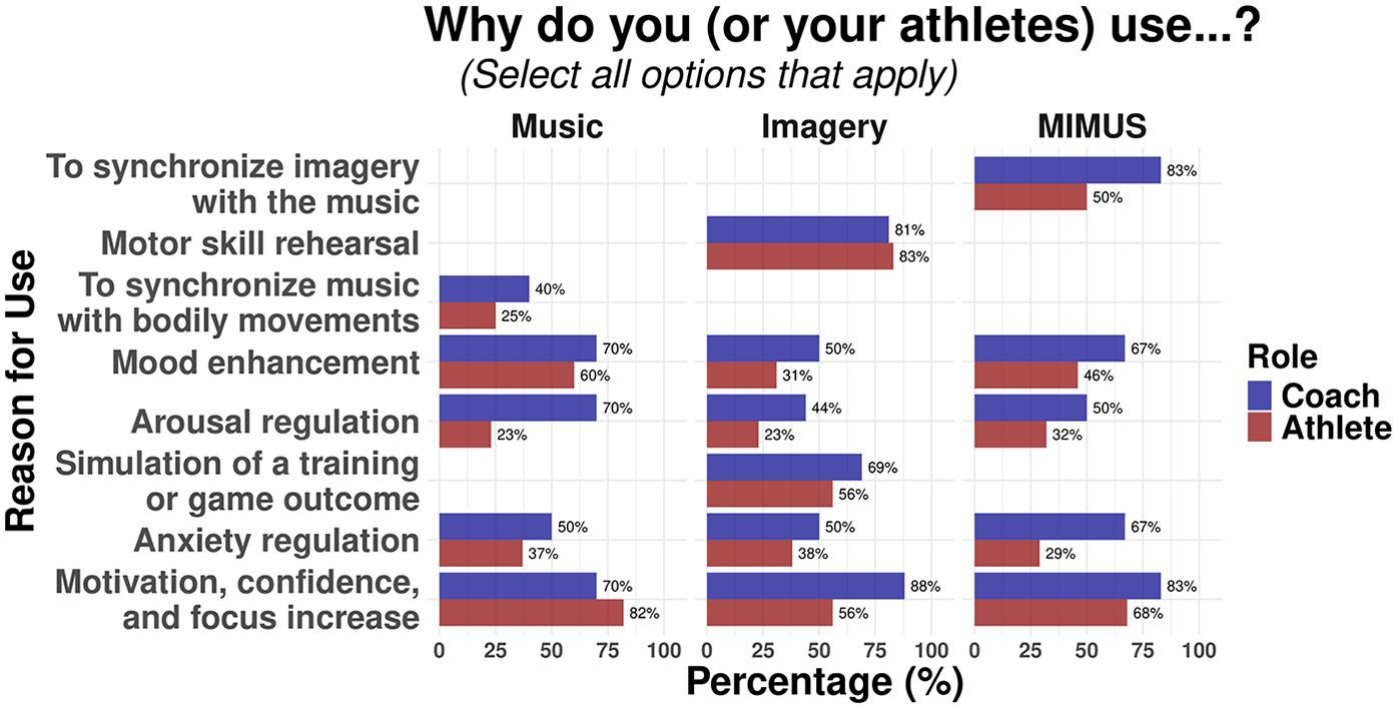
Reasons why athletes and coaches use music, mental imagery, and MIMUS. Note that some questions do not apply to all groups (“Synchronize music with bodily movements” for either imagery or MIMUS) as all three questionnaires appeared at different times. However, all plots were grouped together for visualization purposes.

Further, when asking about underlying motives, participants most used these strategies to enhance motivation, confidence, and focus. This motive was the highest ranked among all participants (Athletes: MUS, 82%; MIMUS, 68%; Coaches: MI, 88%; MUS, 70%; MIMUS, 83%) compared to other motives, except for the use of imagery by athletes, where the rehearsal of a motor skill tops the ranking with 83%. Figure 4 depicts reasons why athletes and coaches use each strategy.

#### 3.5.3 Content

**Figure 5:**
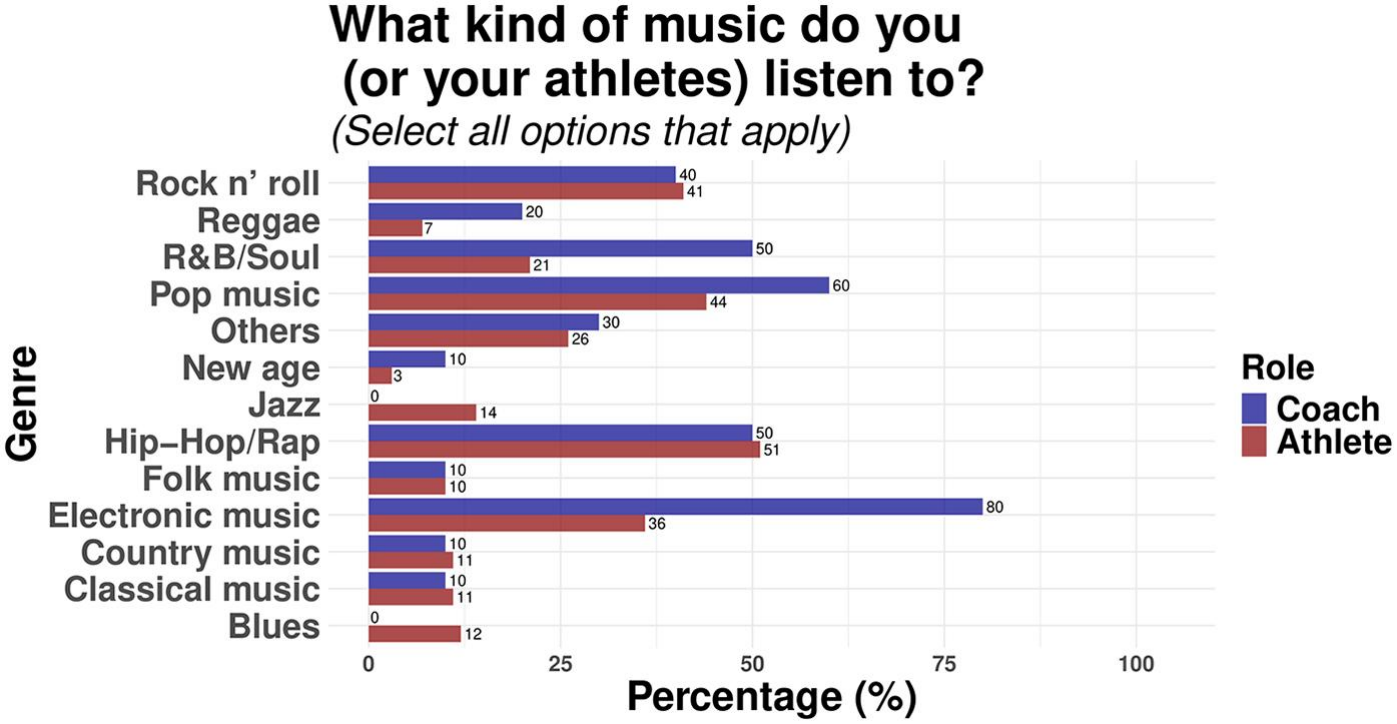
Music genres listened to by coaches and athletes.

**Figure 6:**
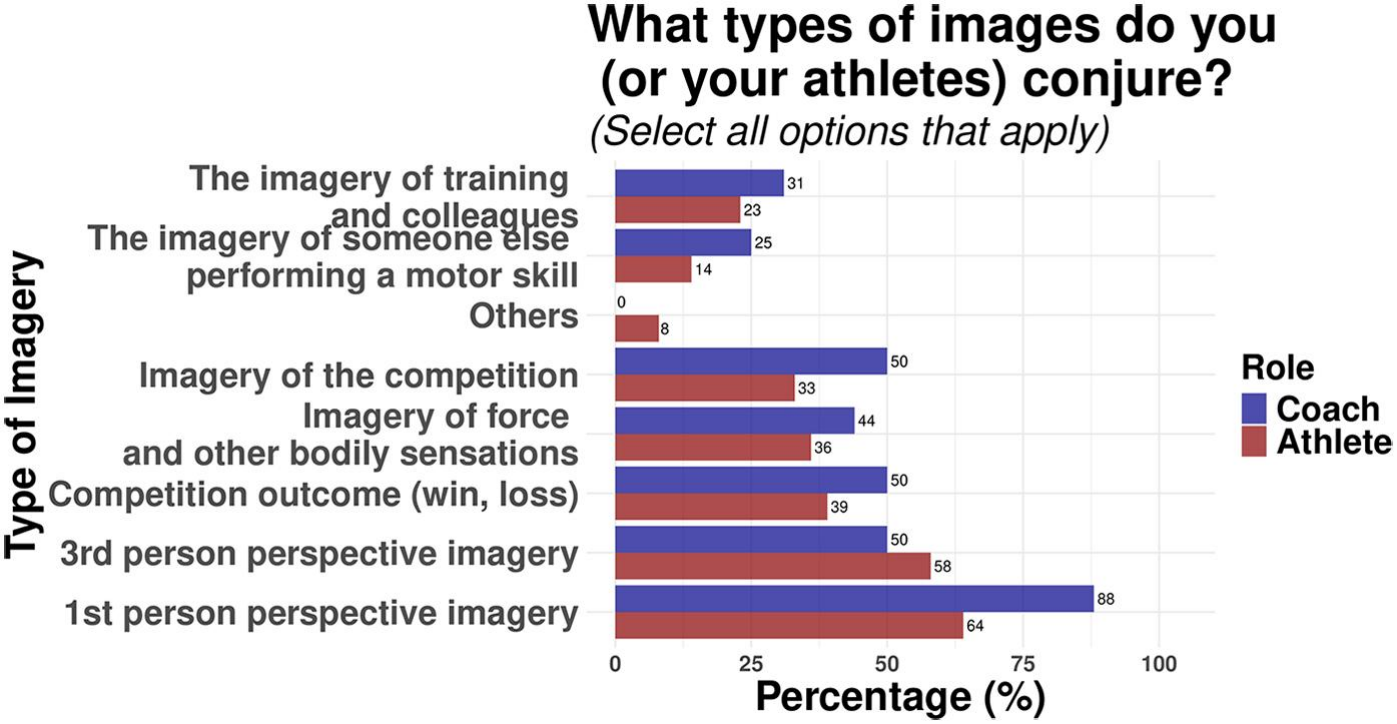
Types of imagery used by coaches and athletes.

Participants also responded to the types of music and mental imagery they used in their training regimen. In terms of music, most athletes (51%) selected hip-hop/rap as their favourite genre, whereas coaches reported frequent use of electronic music in their athlete's routine (80%). Lastly, both athletes and coaches reported a greater use of first-person perspective imagery (Athletes, 64%; Coaches, 88%) compared to other types of mental images. Figures 5, 6 illustrate the type of music and imagery used by participants, respectively.

**Figure 7:**
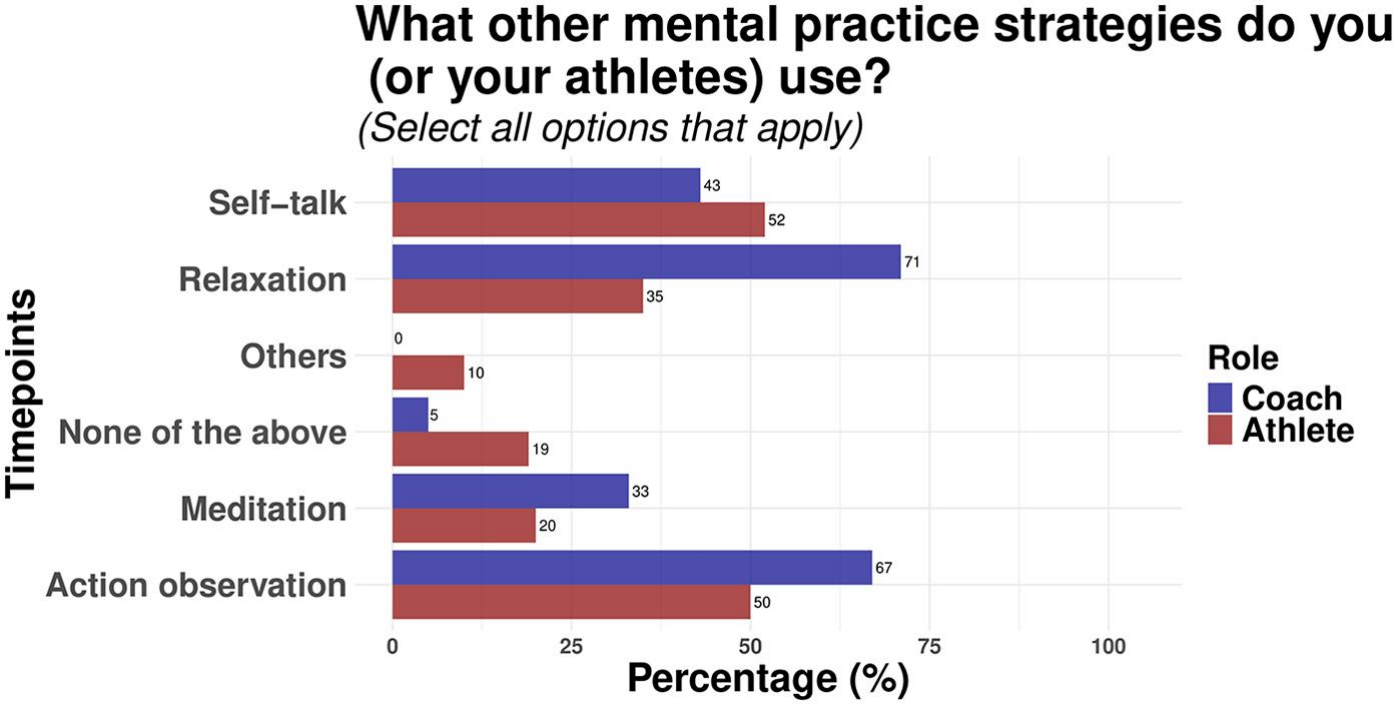
Other mental practice strategies used by athletes and coaches.

Finally, the survey asked participants to indicate what other mental practice strategies they incorporated into their mental training regimen. As shown in Figure 7, the responses varied across athletes and coaches in order of preference. For athletes, self-talk (52%) and action observation (50%) were the highest ranked items, while coaches had relaxation (71%) and action observation (67%) as their preferred mental practice strategies recommended to their athletes.

## 4 Discussion

This study investigated the prevalence of mental imagery, music, and their combination (MIMUS) in a sample of athletes and coaches across the expertise spectrum. We also investigated the contexts in which these strategies are used. Here, we discuss the findings in three key areas: (1) mental imagery, music, and MIMUS form a core part of an athlete's training routine; (2) both samples use the strategies at similar timepoints, but for different reasons; and (3) the strategies are more widely used at the higher tier of the expertise spectrum.

### 4.1 Key finding 1: mental imagery, music, and their combination form a core part of an athlete's mental practice routine

Standalone mental imagery and music had a high prevalence among the whole sample. These results support the expanding role of mental practice as a central pillar of sports psychology, observable via the sharp increase of publications on the effects of mental practice strategies on sports performance in recent years (1, 38). This is also in line with suggestions made in previous studies (15, 16, 39).

Despite being less commonly used amongst the sample, MIMUS is highly used among participants who indicated the standalone use of both mental imagery and music. One explanation is that music is a powerful tool to evoke both goal-directed mental imagery and mind-wandering (25, 40–42), of which both can improve motor skill performance (4, 43). As such, the use of music may trigger mental imagery experiences in athletes, even in cases where this was not the main goal of music listening. The results, however, point out that both coaches and athletes use MIMUS primarily to increase motivation and to synchronize the music with imagined movements, suggesting a goal-directed use of MIMUS.

### 4.2 Key finding 2: both groups use the strategies at similar timepoints, but for different reasons

Like the findings from previous studies (19, 39, 44), athletes and coaches most commonly use all techniques before competition and training sessions rather than during and after the events. On the other hand, the use of imagery during competition was more common than that of music. This finding is not surprising, as most sports governing bodies do not permit the use of music during a competition (38). Although both groups use mental practice strategies in similar timepoints in general, between-group comparisons also paint some interesting observations. First, during-training imagery seems to be far more used by coaches, highlighting a strategic-based approach by this cohort. Conversely, the after-training use of the strategies is more common among athletes than coaches, suggesting that athletes may use them to relieve the physical effects of exercise (38), as well as the use of counterfactual learning (45). Counterfactual learning in sports is the learning from scenarios that did not happen—but could have (46). For example, vividly imagining or simulating alternative game scenarios and their consequences while listening to music, such as “If I had passed the ball instead of shooting it, we could have scored the goal”.

These latter findings are indicative of distinct goals that both athletes and coaches aim to achieve through mental practice. In a seminal conceptual framework (47, 48), the authors lay out the motivational qualities of music (rhythm, musicality, cultural impact, and extra-musical associations) for its use in sports. These qualities, in interaction with moderators such as personal and situational factors, bring about psychological, psychophysical, behavioural, and psychophysiological benefits for the practice of sports (2). For instance, athletes reported higher use of hip-hop and rap than other genres. These genres are known for their motivational factors, with their beats causing a stimulating effect on the listener (38). In line with previous studies (28, 39), our findings also highlight the use of music as, primarily, an emotional regulator. Thus, given its higher use than imagery by athletes in the current study, it is possible to suggest that this cohort may focus more on the regulation of emotions and motivation when engaging in mental practice. This latter suggestion can also be observed in the higher ratings of self-talk reported by athletes—a strategy often used to increase one's motivation in sports (49, 50)—compared to other strategies.

Coaches indicated promoting imagery among athletes to a greater extent than athletes reported employing it themselves. A seminal framework (51) proposes that mental imagery can have both motivational (e.g., imagery related to goals and outcomes and mental toughness), and cognitive (e.g., rehearsal of performance skills and strategies) functions for motor skill performance. Interestingly, coaches indicated that the main use of mental imagery is for motivation, confidence, and focus increase, but responses related to the imagery of a motor skill performance were overall high. In sum, the data is indicative that, on top of using mental practice to regulate their athletes' emotional responses, coaches appear to emphasise strategic and cognitive aspects of the mental practice more than athletes. In addition, 1st person perspective of imagery was reported to be the most preferred choice by coaches by far, reflecting the importance of this perspective in rehearsing the accuracy of performance and motivation (20, 52, 53). Likewise, the higher rating of relaxation reported by coaches suggest a combined use with mental imagery with their athletes*.* As in previous studies (16, 19), but unlike the reported finding that men use imagery more than women (54), this study found no significant gender differences in terms of usage of any mental practice strategy, therefore, no gender-based differences in mental practice strategies could be inferred.

Finally, the use of MIMUS paints a similar picture in terms of goals of mental practice strategies employed by athletes and coaches, as both typically use music in conjunction with imagery to yield higher motivation, but coaches also encourage athletes to use MIMUS for motor skill rehearsal more than athletes reported doing so themselves.

### 4.3 Key finding 3: the strategies are more widely used at the higher tier of the expertise spectrum

Consistent with previous studies (4, 16, 17), participants of international competitive levels report higher use of both music, imagery, or MIMUS than other levels. Interestingly, however, the use of either strategy by national, regional, and recreational participants did not differ significantly and was already fairly high, suggesting an increasing awareness and accessibility of mental training resources even at non-elite levels (4, 20).

Altogether, these findings corroborate the *deliberate practice framework* (3, 5, 55), as well as the role of imagery within it (4). Moreover, imagery is perceived to be a more relevant mental practice strategy for high-skilled athletes than lower-skilled ones, and more cumulative throughout their career (4). Given the paucity of studies investigating the role of music within deliberate practice, the results from this survey suggest that music may also be perceived as a relevant tool in mental practice, and deliberate music listening may also be cumulative in an athlete's career. Future research should examine whether this is the case.

## 5 Implications and future studies

This study offers new perspectives on how athletes and coaches integrate mental imagery, music, and their combination (MIMUS) into training routines, highlighting that these mental practice strategies are not only highly prevalent but also perceived as valuable strategies for psychological and performance enhancement—particularly at the elite level. The findings suggest that mental practice is a key component of mental preparation, valued for its capacity to regulate emotion, enhance motivation, and support motor skill rehearsal. In addition, the fact that coaches and athletes engage with these strategies in distinct but complementary ways further emphasizes their versatility and relevance in sports settings. Crucially, the results show that MIMUS, though less established in the literature, is already being employed strategically, signalling a gap between applied practice and empirical research. Future work should aim to clarify the mechanisms behind this mental practice, determine how it can be optimally integrated into training programs, and assess its long-term effectiveness across different sporting contexts and populations. Finally, future research is warranted to investigate how coaches facilitate and promote the incorporation of these strategies into their athletes' mental practice routines.

This study has a few limitations. First and foremost, the survey was conducted with English-speaking participants only, limiting the generalizability of the findings. Thus, the cultural or regional differences that may influence the use of the strategies across athletes and coaches were not captured by the responses. Also, as the study relied on self-report, the prevalence estimates may be affected due to participants overreporting their engagement with mental practice strategies due to demand biases, or underreported due to possible stigma (39).

In addition, because the survey was anonymous, it is theoretically possible that the same individual could have responded as both an athlete and a coach. However, this is not considered a major limitation in the present study, as responses were provided in relation to the participant's current role, and the demands and perspectives of athletes and coaches are inherently distinct. Finally, despite a reasonable total sample size, results per subgroup should be interpreted with caution given the small sample of each (e.g., recreational-level coaches). These limitations should be considered in future research investigating the prevalence as well as other variables regarding the use of mental imagery, music, and MIMUS in sports.

## 6 Conclusion

This study provides novel insights into the prevalence of mental imagery, music, and their combination as mental practice strategies among athletes and coaches. While usage patterns and motivations vary based on role, both athletes and coaches appreciate their use for emotional regulation as well as motor skill rehearsal. Notably, their increased use among athletes and coaches at the higher end of the expertise spectrum suggests a more widely use of mental training by elite athletes. Finally, by revealing that these practices are already widely used in applied settings—despite limited empirical research (especially on MIMUS)—this study highlights a gap between practice and evidence. Addressing this gap can be essential for developing robust, evidence-based mental training programs in sports.

## Data Availability

The datasets presented in this study can be found in online repositories. The names of the repository/repositories and accession number(s) can be found in the article/Supplementary Material.
