# Supplementary material for: Understanding the prevalence of mental imagery, music, and their combined use among athletes and coaches
Source: Front Sports Act Living. 2025 Oct 13;7:1683432. doi: 10.3389/fspor.2025.1683432 (PMC12554704; doi:10.3389/fspor.2025.1683432)
Supplement: Supplementary file 1 [file Datasheet1.pdf]

List of sports included in the study

Acrobatics

Aerobic Gymnastics

Achery

Arnis

Artistic Gymnastic

Artistic Swimming

Australian Football

Badminton

Baseball

Baton Twirling

Bycicle Motorcross

Billiards/Pool

Bobsleigh

Bodybuilding

Bowling

Boxing

Canoeing

Car Racing

Cheerleading

Chess

Cricket

Croquet

Curling

Dance Sport

Darts

Diving

Dodgeball

Fencing

Figure Skating

Football (U.K)/Soccer(USA)

Frisbee

Golf

Handball

Handgliding

Hockey

Horseback Riding

Horse Racing

Ice Hockey

Ice Skating

Jet Ski Racing

Kayaking

Kendo

Kickboxing

Kitesurfing

Lacrosse

Luge

Martial Arts

Motorcross

Paintball

Parachutting

Polo

Powerlifting

Rafting

Rhythmic Gymnastics

Rowing

Rugby

Sailing

Shooting

Skateboarding

Skiing

Snowboarding

Softball

Speed Skating

Sport Climbing

Squash

Surfing

Swimming

Table Tennis

Tennis

Track and Field

Triathlon

Volleyball
