# Supplementary material for: Understanding the prevalence of mental imagery, music, and their combined use among athletes and coaches
Source: Front Sports Act Living. 2025 Oct 13;7:1683432. doi: 10.3389/fspor.2025.1683432 (PMC12554704; doi:10.3389/fspor.2025.1683432)
Supplement: Supplementary file 2 [file Datasheet2.docx]

**Survey: Exploring the prevalence of music, imagery and these elements combined among athletes**

**Part I - Demographics Questionnaire of Athletes**

Please only respond to this section if you are an athlete. For coaches or sports psychologists, please go to the corresponding section of the survey

**1) Age**

□ Choose an item.

**2) Gender**

□ Choose an item.

**3) Which of the following sports do you professionally practice? Please select one option from the drop-down menu**

□ Choose an item.

**4) How long have you been practising your sport modality professionally?** **Please select one option**

□ Choose an item.

**5) How many hours a week do you practice your sport modality?** **Please select one option**

□ Choose an item.

**6) Please select the option that matches your competitive level.**

□ Choose an item.

**7) Do you have normal hearing or corrected-to-normal hearing (e.g., use of hearing aids)?**

□ Yes □ No

**Part I - Demographics Questionnaire of Coaches**

Please only respond to this section if you are a coach. For athletes or sports psychologists, please go to the corresponding section of the survey

**1) Age**

□ Choose an item.

**2) Gender**

□ Choose an item.

**3) Which of the following sports do you work as a professional coach? Please select one option from the drop-down menu**

□ Choose an item.

**4) How long have you been working as a professional coach?** **Please select one option**

□ Choose an item.

**5) How many hours a week do you coach your athletes?** **Please select one option**

□ Choose an item.

**6) Please select the option that matches the competitive level of the athletes you work with.**

□ Choose an item.

**Part I - Demographics Questionnaire of Sports Psychologists**

Please only respond to this section if you are a sports psychologist. For athletes or coaches, please go to the corresponding sections of the survey

**1) Age**

□ Choose an item.

**2) Gender**

□ Choose an item.

**3) In which of the following sports do you work as a sports psychologist? Please select a minimum of at least one option and a maximum of five options from the drop-down menu**

□ Choose an item.

□ Choose an item.

□ Choose an item.

□ Choose an item.

□ Choose an item.

**3) How long have you been working as a sports psychologist with athletes?** **Please select one option**

□ Choose an item.

**4) How many hours a week do you work with your athletes?** **Please select one option**

□ Choose an item.

**5) Please select the option that matches the competitive level of the athletes you work with.**

□ Choose an item.

**Part II – Survey on mental practice routine in sports (athletes)**

Please only respond to this section if you are an athlete. For coaches or sports psychologists, please go to the corresponding sections of the survey

Thanks for participating in our study *Exploring the prevalence of music, imagery and these elements combined among athletes*. We are interested in finding out about the kinds of mental practices, techniques, and tools that athletes use as part of their training routine. More specifically, we are interested in learning more about the prevalence of the use of music, mental imagery, and these elements combined during your everyday preparation routine. Please answer the questions according to your preferences when it comes to utilizing mental practice techniques and auxiliary tools during your training or competition routine.

**1) Do you use music as part of your mental preparation routine? Select an option.**

□ Yes □ No

**If yes to 1), when do you listen to music? Select all options that apply**

□ Before a training session

□ During a training session

□ After a training session

□ Before competition

□ During competition

□ After competition

**If yes to 1), with what purpose do you use music as part of your preparation routine? Select all options that apply**

□ To feel more motivated, confident, and concentrated before/during/after a training session/competition

□ To regulate my anxiety before/during/after a training session/competition

□ To regulate my arousal levels before/during/after a training session/competition

□ To improve my mood before/during/after a training session/competition

□ To synchronize the rhythm of the music with my body movements or bodily responses to exercise (i.e., heart rate).

**If yes to 1), what are the musical genres that you listen to in your mental preparation routine? Please select as many as you would like**

□ Blues □ Classical music □ Country music □ Electronic music

□ Folk music □ Hip-Hop/Rap □ Jazz □ New age

□ Pop music □ R&B/Soul □ Reggae □ Rock n’ roll

□ Others – Please specify: ____________

**2) Do you use mental imagery techniques in your preparation routine?**

□ Yes □ No

**If yes to 2), when do you use mental imagery techniques? Select all options that apply**

□ Before a training session

□ After a training session

□ Before competition

□ After competition

**If yes to 2), with what purpose do you use mental imagery as part of your preparation routine? Select all options that apply**

□ To mentally rehearse the performance of a motor skill before executing it in a training session/competition

□ To mentally simulate outcomes of a training session/competition

□ To feel more motivated, confident, and concentrated before/after a training session/competition

□ To regulate my anxiety before/after a training session/competition

□ To regulate my arousal levels before/after a training session/competition

□ To regulate my mood before/after a training session/competition

**If yes to 2), what kind of mental images do you conjure? Select all options that apply**

□ The mental imagery of yourself, outside of your body, as if you were watching yourself on a tape, performing a motor skill related to your sport modality.

□ The mental imagery of what you would see as if you were actually playing or performing a motor skill related to your sport modality, in which the image would resemble a headcam shot.

□ The imagery of someone else performing a motor skill related to your sport modality.

□ The mental imagery of the training ground, your teammates, coaches, etc.

□ The mental imagery of the competition venue, your opponents, the crowd, etc.

□ The mental imagery of the outcome of the competition, such as a win, or a loss.

□ The imagery of the feeling of the weight of the equipment or the force you would apply to perform a motor skill related to your sport modality.

□ Others – Please specify: ____________

**3) Do you use mental imagery techniques combined with music in your preparation routine?**

□ Yes □ No

**If yes to 3), when do you use mental imagery and music combined? Select as many options as you would like**

□ Before a training session

□ After a training session

□ Before competition

□ After competition

**If yes to 3), for what purpose? Select as many options as you would like**

□ To synchronize my simulated movements with the music while I mentally rehearse the performance of a motor skill before executing it in a training session/competition

□ To feel more motivated, confident, and concentrated before/after a training session/competition

□ To regulate my anxiety before/after a training session/competition

□ To regulate my arousal levels before/after a training session/competition

□ To regulate my mood before/after a training session/competition

**If applicable, what mental practice techniques do you use as part of your mental preparation routine? Select as many options as you would like**

□ Action observation □ Relaxation □ Meditation □ Self-talk

□ Others (please specify): _____________ □ None of the above

**If applicable, what tools do you use as part of your mental preparation routine? Select as many options as you would like**

□ Reading sports-related content

□ Watching sports-related material

□ Listening to audiobooks, motivational speeches, sounds, related to sports etc.

□ Accessing social media or other platforms to obtain information related to sports

□ Reading/Watching/Listening content **not** related to sports

□ None of the above

**Part II – Survey on mental practice routine in sports (coaches and sports psychologists)**

Please only respond to this section if you are a coach or a sport psychologist. For athletes, please go to the corresponding section of the survey

Thanks for participating in our study *Exploring the prevalence of music, imagery and these elements combined among athletes*. We are interested in finding out about the kinds of mental practices, techniques, and tools that coaches and sports psychologists use with their clients/athletes as part of their mental training routine. More specifically, we are interested in learning about the prevalence of the use of music, mental imagery, and these elements combined during an athlete’s preparation routine. Please answer the questions according to your preferences when it comes to utilizing mental practice techniques and auxiliary tools with your clients/athletes during their training or competition routine.

**1) Do you use music as part of your athletes’ mental preparation routine? Please select an option.**

□ Yes □ No

**If yes to 1), when do you ask them to listen to music? Select all options that apply**

□ Before a training session

□ During a training session

□ After a training session

□ Before competition

□ During competition

□ After competition

**If yes to 1), with what purpose do you use music as part as the preparation routine of your athletes? Select as many options as you would like**

□ To make them feel more motivated, confident, and concentrated before/during/after a training session/competition

□ To regulate their anxiety before/during/after a training session/competition

□ To regulate their arousal levels before/during/after a training session/competition

□ To regulate their mood before/during/after a training session/competition

□ To synchronize the rhythm of the music with their bodily movements or bodily responses to exercise (i.e., heart rate, inspiration rate).

**If yes to 1), what are the musical genres you suggest your athletes to listen as part of their mental preparation routine? Please select all options that apply**

□ Blues □ Classical music □ Country music □ Electronic music

□ Folk music □ Hip-Hop/Rap □ Jazz □ New age

□ Pop music □ R&B/Soul □ Reggae □ Rock n’ roll

□ Others – Please specify: ____________

**2) Do you use mental imagery techniques in the mental preparation routine of your athletes?**

□ Yes □ No

**If yes to 2), when do you ask them to use mental imagery techniques? Select as many options as applicable**

□ Before a training session

□ After a training session

□ Before competition

□ After competition

**If yes to 2), with what purpose do you use mental imagery as part of the preparation routine of your athletes? Select all that apply**

□ To help them to mentally rehearse the performance of motor skills before executing them in a training session/competition

□ To help them to mentally simulate the outcomes of a training session/competition

□ To make them feel more motivated, confident, and concentrated before/after a training session/competition

□ To regulate their anxiety before/after a training session/competition

□ To regulate their arousal levels before/after a training session/competition

□ To regulate their mood before/after a training session/competition

**If yes to 2), what kind of mental images do you ask them to conjure? Please select as many as you would like.**

□ The mental imagery of themselves, outside of their body, as if they were watching themselves on a tape, performing a motor skill related to their sport modality.

□ The mental imagery of what they would see as if they were actually playing or performing a motor skill related to their sport modality, in which the image would resemble a headcam shot.

□ The imagery of someone else performing a motor skill related to their sport modality.

□ The mental imagery of the training ground, the teammates, coaches giving instructions, etc.

□ The mental imagery of the competition venue, their opponents, the crowd, etc.

□ The mental imagery of the outcome of the competition, such as a win, or a loss.

□ The imagery of the feeling of the weight of the equipment or the force they would apply to perform a motor skill related to their sport modality.

□ Others – Please specify: ____________

**3) Do you use mental imagery techniques combined with music in the preparation routine of your athletes?**

□ Yes □ No

**If yes to 3), when do you use mental imagery and music combined in the mental preparation routine of your athletes? Select as many options as you would like**

□ Before a training session

□ After a training session

□ Before competition

□ After competition

**If yes to 3), for what purpose? Select as many options as you would like**

□ To synchronize their simulated movements with the music while they mentally rehearse a movement before executing it in a training session/competition

□ To help them to feel more motivated, confident, and concentrated before/after a training session/competition

□ To regulate their anxiety before/after a training session/competition

□ To regulate their arousal levels before/after a training session/competition

□ To regulate their mood before/after a training session/competition

**If applicable, what mental practice techniques do you use as part of the mental preparation routine of your athletes? Select as many options as you would like**

□ Action observation □ Relaxation □ Meditation □ Self-talk

□ Others (please specify): _____________ □ None of the above

**If applicable, what tools do you use as part of the mental preparation routine of your athletes? Select as many options as you would like**

□ Reading sports-related content

□ Watching sports-related material

□ Listening to audiobooks, motivational speeches, sounds, etc.

□ Accessing social media or other platforms to obtain information related to sports

□ Reading/Watching/Listening content **not** related to sports

□ Others (please specify): _____________

□ None of the above
